# Supplementary material for: Are carbon emissions trading and green financial instruments synergistic? -Comprehensive quantitative research based on content analysis
Source: PLoS One. 2024 Mar 7;19(3):e0298601. doi: 10.1371/journal.pone.0298601 (PMC10919601; doi:10.1371/journal.pone.0298601)
Supplement: S1 Data — (ZIP) [file pone.0298601.s001.zip › Data/地级市Z_F工作报告.zip!/╔┬╬≈/╬≈░▓╩╨/└┤╫╘╣1⁄2╓┌║┼í╛┬φ┐╦╩2╛▌═°í┐_2015.docx]

各位代表：

现在，我代表市人民政府向大会作工作报告，请予审议，并请市政协委员和其他列席人员提出意见。

一、2014年工作回顾

过去一年，面对极为复杂严峻的经济形势，我们在省委、省政府和市委的坚强领导下，认真贯彻落实党的十八大、十八届三中、四中全会和习近平总书记系列重要讲话精神，坚持稳中求进、改革创新、提质增效的工作总基调，凝心聚力、迎难而上、真抓实干，较好地完成了市十五届人大四次会议确定的主要任务。总体呈现以下特点：

——经济运行稳中向好。全市生产总值突破5000亿元大关，达到5474.8亿元，增长9.9%，分别高出全国和全省2.5和0.2个百分点，增速位居15个副省级城市第二。人均GDP首次超过1万美元，达到10394美元。财政总收入迈上千亿元台阶，较2010年实现翻番。一般公共预算收入完成583.8亿元，增长16.3%。城镇登记失业率3.4%，物价上涨1.4%，明显低于控制目标。

——结构调整步伐加快。战略性新兴产业增加值增长16.8%，高出生产总值增速6.9个百分点。服务业增加值完成3054.9亿元，增长9%，高出全省0.6个百分点。非公经济占生产总值的比重达到52.7%，较上年提高0.5个百分点。万元GDP能耗下降4%左右，单位GDP建设用地下降8.5%。13项指标提前超额完成“十二五”规划目标。

——功能优势竞相释放。19项国家级试点、示范城市建设不断取得新进展，新能源汽车产销量位居全国第一，跨境贸易电子商务服务试点平台上线运行。我市一批重点工作成为全国亮点，被评为2014年度“中国领军智慧城市”、“中国质量魅力城市”和“中国最具文化软实力城市”。

——对外开放亮点纷呈。丝绸之路经济带新起点建设实现良好开局，国家一类陆路口岸开放正式获批，西安高新综合保税区封关运行，丝路联合申遗取得成功，我市5处遗迹入选世界文化遗产。中俄丝绸之路高科技产业园区签约落户。三星项目累计引进配套企业74家。全年进出口总额增长37.7%，增速位居全国同类城市第一，外贸依存度较上年提高5.4个百分点，荣登联合国“全球最具发展潜力新兴城市”榜单。

——城市治理成效明显。铁腕治霾再创佳绩，6项空气主要污染物年均浓度大幅下降，全年二级以上良好天数达到211天，较上年增加73天，如期退出全国重点监测城市空气质量排名后十位。渭河西安段“三年变清”目标顺利完成，氵皂河污染治理实现达标。缓堵保畅三年行动圆满“收官”，群众满意率达到84.1%。100条“标准化管理示范街”展现新姿。南门地区综合改造被评为全国历史文化名城建设重大示范工程，成为美丽西安的新名片。

——民生福祉持续改善。“十项惠民实事”全面落实。城乡居民收入分别达到36100元和14462元，增长9.1%和11.9%。出台改善城区困难群众生活“22条措施”，使23.5万困难群众受益。完成了3.2万移民搬迁和21.3万农村人口饮水安全任务，14.9万人实现脱贫。连续三年荣获“中国最具幸福感城市”称号。

一年来，我们主要做了六个方面工作：

（一）着力深化改革，激发市场活力。根据市委的统一部署，我们坚持以问题为导向全面深化改革，年度涉及政府改革的172项具体任务，已完成162项，改革红利在更多领域加速释放。

坚持下好简政放权“先手棋”。制定公布市级行政审批目录，分批取消和下放了136项行政事权。扎实推进“营改增”扩围试点和工商登记制度改革，为小微企业减免退税92.5亿元，全年新增私营企业户数和注册资本分别是上年的1.8倍和5.4倍，呈现“井喷”之势。

狠抓重点领域改革。启动了新一轮国资国企改革，混合所有制试点有序推进，西纺集团与五环集团完成重组整合。农村土地确权登记工作全面展开，累计流转土地48.8万亩。深入推进财政投入方式改革，通过建立政府增信、风险补偿、奖励补助等机制，有效调动了金融机构支持企业发展的积极性，全年新增中小微企业融资684.7亿元，增长22.9%。持续加大统筹科技资源改革力度，全市研发经费投入强度达5.3%，高出全国3.2个百分点，全年认定科技小巨人企业430家，实现技术成果交易额530.5亿元，增长27.6%，科技创新对经济增长的贡献率达到58.3%。

（二）着力强本固基，加速工业突破。认真落实省政府促进工业稳增长的若干意见，深入实施工业突破战略，全市规模以上工业增加值完成1195.3亿元，增长11.1%。

以百项重点工业项目为抓手，不断夯实工业发展基础。全年安排市级工业专项资金6.6亿元，是2011年的3.3倍。比亚迪二期等71个重点工业项目建成投产，全市工业投资增长38.8%，分别高出全国和全省25.9和30.6个百分点。新增规模以上工业企业153户，超额完成年度目标任务。

以渭北工业区建设为重点，积极培育新的增长极。渭北大横线、泾河引水等50个重点基础设施项目进展顺利，中国黄金、大安丝束等重大产业项目建成投产，新舟700正式签约落户，区内规模以上工业增加值完成261.3亿元，增长13.4%，高于全市2.3个百分点。

以战略性新兴产业为引领，全力打造西安工业升级版。三星闪存芯片项目如期量产，国内最大的汽车动力电池生产基地三星SDI项目和中兴1500万部智能手机项目开工建设。软件新城（一期）建成运营，全市软件产业实现业务收入910亿元，增长30.8%。

（三）着力稳定增长，提高质量效益。适时出台了稳增长“36条”等一系列政策措施，突出重点，狠抓落实，有力保障了经济平稳较快增长。

坚持把稳投资作为稳增长的关键举措。抓住中、省加强基础设施建设的政策机遇，启动实施了市域轨道交通、火车站改扩建等一批重大项目，552个市级重点项目共完成投资2217.5亿元，占年计划的121.4%，其中399个产业类项目完成投资1337.6亿元，是上年的1.3倍。全年完成城建投资352.8亿元，增长18.5%。鼓励支持民营资本进入六大公共领域，全年吸纳民间投资3082.5亿元，占全市比重52.9%。西北首个民间金融街正式运营。我市被评为“2014中国十大区域性金融中心城市”。

坚持把扩消费作为稳增长的基础动力。促进传统商贸餐饮业转型升级，沙河水街、永兴坊特色美食商业街区建成开业。电子商务、健康养老成为新的消费热点，京东、阿里巴巴、唯品会等知名电商企业落户运营，电商交易总额达到1200亿元。全年新增限额以上商贸企业208户，完成社会消费品零售总额2872.9亿元，增长12.8%。有效发挥公积金在促进住房消费中的保障作用，12万职工利用公积金贷款改善了住房条件，带动房地产市场消费500多亿元。制定出台旅游业持续健康发展“新10条”，全年接待海内外游客1.2亿人次，实现旅游总收入950亿元，入选“年度国际游客满意度最高中国旅游城市”，再次荣获“最佳国内旅游城市奖”。成功举办首届丝绸之路经济带国际博览会、西部文化产业博览会等大型展会175个，成交额达1207.5亿元，被评为“2014中国十大影响力会展城市”。

坚持把促开放作为稳增长的重要手段。全面启动丝绸之路经济带新起点建设，欧亚经济论坛综合园区等19个重点合作项目加快实施，“西安港”建设稳步推进，“长安号”实现常态运行。加强与丝路沿线国家、城市互联互通，与土库曼斯坦马雷市、亚美尼亚久姆里市正式缔结友城关系，成为西北首个72小时过境免签城市。全年实际利用外资37亿美元，实际引进内资1692.8亿元，分别增长18.3%和18.4%。

（四）着力优化环境，建设宜居城市。顺应群众对优美舒适生活环境的新期待，我们坚持把宜居作为城市建设管理的最高标准，下决心解决了一批市民反映强烈的“老大难”问题，城市综合功能和品质形象进一步提升。

持续增强城市综合承载力。编制完成了《城市设计导则》、《生态隔离体系控制规划》等一批约束性规划，基本划定城市生态边界。缓堵保畅工作取得阶段性成果，三年累计完成投资488.6亿元，地铁通车里程达到52公里，环城南路综合改造、太白—丈八立交等15项重大交通工程建成投用，新建公交港湾331处、公共停车位2万个，投放公共自行车3万辆，公交机动化出行分担率提高至56%。天然气城市气化（二期）、电网改造、水源建设等一批重点基础设施工程扎实推进，新增集中供热面积2005万平方米；新建和增容改造110千伏变电站11座，增加变电容量74万千伏安；李家河引水系统实现向城区供水，新增城市日供水能力21万吨；城镇污水处理率达到94.5%，在全国同类城市中位居前列。

推动美丽西安建设向纵深拓展。“治污减霾·保卫蓝天”行动扎实有效，全年投入财政资金31.3亿元，是上年的2.1倍，共拆除燃煤锅炉480台，淘汰黄标车及老旧车4.2万辆，对3.4万户居民实施了清洁能源改造，人工影响天气作业实现常态化，全市优良天数超出省考指标71天，群众满意度较上年提高17.7个百分点。全力做好秦岭生态保护，高标准建成8个生态节点广场和17公里环山绿道，全年新增城市绿地面积450万平方米，造林11.5万亩。加快推进八水润西安工程，完成“两河五湖六湿地”建设，新增生态水面3108亩、湿地6506亩。积极开展全国文明城市创建活动，大力整治环境卫生、市容秩序、集贸市场等突出问题，诸多群众关切的城市管理热点、难点问题得到有效解决。

更加注重城镇功能的完善提升。按照“一类一策”、“分类推进”的原则，持续加大对区县的支持力度，3个副中心城市、5个城市组团和13个重点镇建设步伐加快。启动实施了49个农村片区化中心社区和325个新农村重点村建设，一批高标准、现代化的农村商贸、文体、卫生等基础设施建成投用。高陵撤县设区，并被列为全国首批新型城镇化综合试点。全年接收18.4万有条件的农村居民进城落户，城镇化率达到72.6%，较上年提高0.6个百分点。都市型现代农业“服务城市、富裕农民”的功能进一步增强，新增省、市级现代农业示范园36个，全市粮食总产量达到175.6万吨，实现“十一连丰”。

（五）着力改善民生，促进社会公平。全市一般公共预算支出的81.7%和新增财力的83.9%用于民生，总计达到669.4亿元，增长15.2%，广大群众从改革发展中得到了越来越多的实惠。

以“十项惠民实事”为抓手，大力实施民生工程。全年城镇新增就业13.2万人，转移农村劳动力95.3万人。新开工保障性安居工程10.3万套，提供房源4.4万套，改造农村危房1.1万户。完成了27个城棚改项目征收任务，回迁安置群众3.9万人。深入治理“餐桌污染”，改造提升“小餐饮”1.4万户，创建放心食品点、蔬菜直销店225个。建成农村幸福院200个、社区养老服务设施80个，全市养老床位达到3.2万张。按照城乡统一标准，将居民养老保险基础养老金提高至每人每月130元，受益群众超过60万。

以促进社会和谐为目标，推动社会事业全面进步。大学区管理制改革深入实施，36所开发区学校、幼儿园建设加快推进，130所义务教育学校标准化建设如期完成，营养改善计划惠及学生38.3万人。卫生服务能力有效提升，市中医医院完成搬迁，全面实施了家庭医生签约式服务。城镇职工慢性病补助限额标准平均上调15%，城乡居民医保财政补助标准提高至每人350元，新农合参合率和城镇基本医疗保险参保率分别达到99.2%和98.5%。计划生育“单独两孩”政策平稳落地，“母亲健康工程”和“优生促进工程”惠及群众61.5万人。投入2270万元开展“千场戏剧”惠民演出和公益性电影放映，建成广播电视“户户通”工程8.3万户，新建农民健身工程760个、社区全民健身路径120个，群众性精神文明创建活动不断取得新成效。

以创新社会治理为基础，全力维护社会稳定。推行社区网格化管理，大幅提高社区工作经费，老旧小区改造取得新进展。基层公共法律服务平台建设富有成效，13个区县公共法律服务中心全部建成投用。“平安西安”建设深入推进，建立完善了“四位一体”反恐工作机制，三环内68个警务站投入使用。有效化解信访积案，全市信访化解率较上年提高9个百分点。常态化开展安全生产大检查，强化道路交通、建筑施工、危化品等重点行业领域专项整治，成功处置了“5·26”液化气罐车泄漏等突发事件，全年未发生重特大事故。

（六）着力正风肃纪，推进依法行政。市政府班子党的群众路线教育实践活动整改措施全面落实，及时解决了一批群众反映的突出问题。市级“三公”经费信息全面公开，全市“三公”经费同比下降18.3%。制定了市级行政审批“权力清单”，引入第三方评价政府绩效，撤销议事协调机构228个。自觉接受人大及其常委会的依法监督和政协民主监督，认真办理人大代表建议、意见和政协提案，办复率达100%。认真落实“两个责任”和“一岗双责”，不断加大治慵惩腐力度，政府系统廉政建设和反腐败工作取得了新的明显成效。

过去一年，我市双拥共建、民族、宗教、参事、人防、史志、档案、防震减灾等领域的工作也都取得了新的成绩。

各位代表！回顾过去一年的工作，我们遇到的困难比预料的多，承受的压力比预想的大，但我们始终坚持低调务实不张扬，埋头苦干，锐意进取，取得了来之不易的成绩，也干成了一批事关长远发展和百姓福祉的大事、要事。这是市委坚强领导的结果，是市人大和市政协监督支持的结果，是全市上下团结奋斗的结果。在此，我代表市政府，向全市人民，向中、省驻市单位，向驻西安部队、武警官兵和公安干警，向所有关心支持西安建设发展的海内外朋友，表示衷心的感谢！

在总结成绩的同时，我们也清醒地看到，工作中还存在不少差距和问题，主要表现在：一是长期制约经济发展的深层次矛盾和问题仍未根本解决，经济总量依然偏小，总体发展水平与国内发达地区特别是同为西部城市的成都相比差距较大，稳中求进、动力转换、提质发展的要求更加迫切；二是受宏观经济影响和资源环境约束，稳增长的压力持续加大，转方式、调结构、促升级、防风险的任务十分艰巨，已进入爬坡过坎、转型跨越的关键阶段；三是政府职能转变还不到位，市场监管、城市治理和公共服务能力有待加强，法治政府建设和反腐败斗争仍是长期任务。对此，我们一定要采取切实措施，认真加以解决。

二、新常态下经济社会发展的基本要求和2015年主要指标

当前，宏观经济环境正在发生深刻变化，中央作出了我国经济发展进入新常态的科学论断。保持中高速增长、迈向中高端水平，既是新常态的发展必然，也是今后工作的基本遵循。

综观新常态下西安经济发展的趋势性变化，可以作出这样三个判断：虽然我市经济发展进入调速换挡期，但稳中向好的基本面依然没有改变；虽然长期积累的深层次矛盾和新出现的问题相互交织，但保持经济中高速增长的势能依然比较充足；虽然经济运行中不稳定不确定因素增多，但支撑经济发展的机遇和优势依然比较明显。因此，我们必须准确把握发展大势，正确认识和主动适应新常态，对标先进，自我加压，积极作为，全面加快建设具有历史文化特色的国际化大都市，努力当好“三个陕西”建设的排头兵。

要更加坚定发展是第一要务。始终把稳增长放在经济工作的首位，坚持底线思维，保持发展定力，既要稳住速度，确保经济运行在合理区间，又要提质增效，确保经济持续健康发展，努力做到调速不减势、量增质更优。

要更加注重经济结构优化升级。始终把高端化作为结构调整的主攻方向，按照“四化同步”的要求，坚持“二产与三产并举、传统与新兴并重”，积极构建与国际化大都市建设相适应的现代产业体系，努力打造西安经济升级版。

要更加突出改革创新的引领支撑作用。始终把全面深化改革作为西安高端高质发展的根本动力，不断强化创新驱动，积极推进高水平对外开放，持续激发市场活力、增强发展内生动力，努力在新常态下推动西安发展迈上新台阶。

要更加关注民生改善和社会和谐。始终把改善民生作为正确处理改革发展稳定关系的结合点，按照补短兜底、完善机制、强化保障的思路，统筹推进各项社会事业协调发展，着力增加公共产品有效供给，努力提高全市人民的幸福指数。

2015年，政府工作的总体要求是：全面贯彻落实党的十八大、十八届三中、四中全会和习近平总书记系列重要讲话精神，坚持稳中求进、改革创新、依法行政的工作基调，以建设丝绸之路经济带新起点为统领，以提高经济发展的质量和效益为中心，持续推进“五项重点工作”，突出抓好“十项清零行动”，全力稳定增长，加快调整结构，积极扩大开放，狠抓城市治理，不断改善民生，确保经济平稳健康发展和社会和谐稳定。

主要预期目标是：生产总值增长10.5%左右，一般公共预算收入可比增长13%，城乡居民收入分别增长10%以上和12%左右，城镇登记失业率控制在4%以内，物价涨幅控制在3%左右，万元GDP能耗下降3.2%，万元GDP二氧化碳排放下降3.7%。以上安排，既是顺利实现全面建成小康社会目标的需要，也是在全省发挥支撑和引领作用的要求；既符合我市发展实际和民生需求，也为深入调整结构、促进转型升级留有一定余地；同时也是稳定市场预期、提振社会信心，全面完成“十二五”规划的基本保证。因此，我们不能有丝毫懈怠，必须凝神聚力谋发展，心无旁骛抓落实，努力在新常态下实现西安发展的新跨越。

三、2015年的主要工作任务

（一）全力以赴稳增长，确保经济平稳运行在合理区间。

充分发挥投资关键作用。继续实施项目带动战略，确保500个市级重点建设项目年度完成投资1820亿元以上，全年完成城建投资388亿元，力争全社会固定资产投资增长20%左右。着力优化投资结构，充分发挥财政投资在民生改善、公共服务、基础设施互联互通等方面的引导作用，不断提升产业投资比重，加强技术改造、科技创新等领域的投资。切实增强社会投资活力，推动社会资本进入基础产业、金融服务、教育医疗、水利建设等领域，力争民间投资占比达到55%以上。进一步做好招商引资工作，狠抓重大招商签约项目的跟踪落实，全年实际利用外资和引进内资分别达到40亿美元和1680亿元。扎实推进多层次资本市场建设，积极推广PPP投资模式，全年新增上市和“新三板”挂牌企业10家以上。

不断夯实消费基础作用。多渠道增加居民收入，深入实施农民收入倍增计划，坚决落实居民收入与经济增长和财政收入增长联动机制，落实职工带薪休假制度，切实增强居民消费能力。持续扩大新能源汽车消费，稳定住房消费，大力培育信息、休闲、健康、养老等新兴消费，促进个性化、多样化消费，不断提高服务性消费在居民消费支出中的占比。加快太华路、大兴新区等新兴商圈建设，推动商业设施向地铁沿线和城市新区布点，完善社区、农村便民商业服务网络，不断提升消费承载力。加强市场监管，依法严厉打击制售假冒伪劣产品、价格欺诈等不法行为，加大消费维权力度，保持物价总水平稳定，全社会消费品零售总额增长13%左右。

着力增强企业支撑作用。围绕“龙头企业强实力、小微企业增活力”两个重点，整合各类扶持资金，采取资本金投入、有偿使用、贷款贴息、以奖代补等方式，优先向重点企业、薄弱环节进行投放，帮助企业勇闯难关，不断做大做强。继续完善政府、银行、担保机构和企业共同参与的“四方合作”机制，有效缓解企业“融资难、融资贵”问题。大力培育各类市场主体，促进百企成长、万众创业，力争全年新增规模以上工业企业100户、限额以上贸易企业60户、规模以上服务业企业200户，全市工商注册企业突破18万户。

进一步强化创新驱动作用。支持高新区创建国家自主创新示范区，加快推进“一带一城四集群”建设。强化企业创新主体地位，围绕产业链布局创新链，建立以市场为导向、以产业化为目标的科技创新管理体制，完善市校合作、研企互动的协同创新机制，引导优势科技资源向产业聚集，力争全年技术成果交易额突破650亿元、就地转化率达到30%以上。加快实施培育科技企业小巨人三年行动方案，年内打造领军企业40家，力争全市科技小巨人企业超过700家，高新技术产业增加值增长12.5%左右。

（二）突出重点抓改革，不断增强发展内生动力。

继续深化行政管理体制改革。持续加大简政放权力度，严格实施行政审批“权力清单”，年内再取消下放一批市级行政审批事项。完成市、区县政府机构改革，推进开发区管理体制机制创新，全面实行机构编制实名制管理。进一步深化财税体制改革，完善全口径预算体系，将新举借政府性债务纳入预算，全市各级使用财政资金的部门除涉密信息外全面公开预决算。年内完成公车改革。

深入推进市场化改革。坚持非禁即入放宽民营经济发展领域，6月底前制定公布市场准入“负面清单”，着力消除对非公有制经济的各种隐性壁垒。不断优化营商环境，深化商事登记制度改革，强化事中事后监管。加快推进社会信用体系建设，健全完善“黑名单”制度，大幅提高企业违法失信成本。全面完成农村土地承包经营权确权登记，引导和规范土地经营权有序流转，逐步把沉睡的资产变成流动的资本。

切实抓好国资国企改革。落实市属国企改革“1+N”方案，推进混合所有制试点，加快国有资产证券化步伐，进一步理顺国有资产监管体制机制。加快五环集团搬迁入园进度，力争西安纺控新厂区项目全面投入运营。

（三）转型升级调结构，推动产业发展向高端化迈进。

继续深入实施工业突破战略。全年规模以上工业增加值增长12%左右，工业投资增长稳定在30%以上。全面加快渭北工业区建设。抓住高陵撤县设区的机遇，扎实推进工业区基础设施配套，年内渭北大横线全线贯通。统筹做好西安至阎良快速干道、城际铁路等重大交通项目的各项准备工作，力争年内开工建设。积极争取延长石油、西北轨道交通产业基地等重大项目签约落户，主动对接西电集团整体搬迁，确保区内规模以上工业增加值增长13%以上。大力发展战略性新兴产业。突出抓好三星电子、中兴手机、新舟700、新能源汽车和强生供应链基地等“旗舰”项目，不断完善产业链条，增强聚集优势，着力打造半导体、智能终端、航空航天、生物医药等一批千亿元产业集群。积极引导主城区工业企业退城入区、转型发展，年内完成搬迁企业14户。加快国家下一代互联网示范城市和智慧城市建设，运用信息技术改造提升传统制造业，推进设计研发信息化、生产装备数字化和生产过程智能化，全面提升我市工业的综合竞争力。

推动服务业优化提升。按照“传统与新兴并重”的思路，促进传统产业加快升级、新兴产业加快成长，力争全年服务业增加值增长10%左右。创新发展互联网内容产业，进一步拓展大数据、云计算、物联网等示范应用，推动电子商务与新型流通业态深度融合。支持传统商贸企业发展线上业务，促进电商服务向农村延伸覆盖，力争全年电商交易总额突破1500亿元。加快推进西安金融商务区、民间金融街（二期）等金融服务业聚集区建设，支持互联网金融等新兴业态健康发展，严厉打击各类非法集资行为，营造良好金融生态环境。大力发展检测检验、工程咨询、创业孵化等综合科技服务，不断提升科技服务业对产业发展的支撑能力，全年科技服务业增加值达到600亿元。协力办好欧亚经济论坛、中国国际通航大会等大型展会活动，加快构建具有国际影响力的会展产业集群。加速传统服务业转型升级，鼓励开发多种模式的城市商业综合体，促进商贸、餐饮、旅游、休闲等服务业态聚合发展，继续抓好东大街、解放路等传统商圈改造提升，加快推进长安路、唐延路等现代商务聚集区建设，着力打造秦岭北麓环山健康休闲产业带。深入整治旅游市场秩序，创新开展旅游宣传营销，加快航空大世界、丝绸之路风情街（二期）等25个重点旅游文化项目建设，不断提升城市旅游综合服务功能，力争全年接待海内外游客1.36亿人次，旅游总收入达到1070亿元。加快西安中央文化商务区、雁翔路国家级文化产业聚集区和西安设计创意产业园建设，推进文化产业与旅游、科技、金融等产业融合发展，精心打造一批国家级大剧大作，着力提升我市文化产业的整体实力和竞争力，全年文化产业增加值增长11%以上。

大力发展都市型现代农业。以提高农业组织化、市场化、品牌化水平为重点，加快推进“一区三带七板块”建设，年内认定扶持市级以上现代农业园区25个。积极实施农业提质增效“五大工程”，新建粮食高产万亩示范片51个、设施蔬菜日光温室2500亩，改扩建标准化规模养殖场100个，新增果业面积2万亩、市级以上休闲农业园区10个。加快培育新型农业经营主体，组织开展农民专业合作社“十佳示范社”评选活动，年内认定市级优秀示范社20家、示范家庭农场10个。

（四）立足优势促开放，着力打造内陆开放新高地。

全面加快“新起点”建设。编制完成《丝绸之路经济带新起点发展规划》。扎实做好西安自贸区申报工作。举全市之力争取中国与新加坡政府合作项目签约落户。加快推进西安领事馆区、中西部大宗商品交易中心等32个重点项目建设。进一步增强“长安号”营运能力，规划建设新筑综合性货场，全面提升“西安港”综合服务功能，力争年内纳入国际贸易运输体系。建立陆港与空港联动发展机制，加快建设快速货运连接线，年内争取开通西安至阿拉木图等8条国际客运、货运航线，打造“立体物流”新模式。

不断优化对外开放环境。完善丝绸之路经济带沿线城市合作发展圆桌会议机制，加快落实各项合作协议，巩固深化能源、金融、教育、旅游等领域的务实合作，积极拓展高新技术、现代农业、生态环保等新的合作领域。加强对丝路沿线重点国家政策、法律等方面的研究，编制《中亚五国投资指南》，为西安企业“走出去”提供帮助和指导。以加快建设国际学校、医院和社区为重点，全面提升城市涉外公共服务水平，为外籍人士在西安工作、学习、生活提供良好保障。

积极争创对外开放新优势。加快电子口岸建设，强化跨境贸易结算、离岸金融、贸易金融等功能，探索开展保税进口和直购进口业务，推动外贸发展方式转变。完善综合保税区配套设施建设，继续抓好展示交易、保税物流、出口加工、服务外包等业态发展，不断优化对外贸易结构。力争全年进出口总额达到215亿美元，服务外包合同金额增长28%以上。

（五）统筹城乡强功能，加快新型城镇化建设步伐。

进一步优化城市空间格局。认真做好第四轮城市总体规划修改和“多规合一”试点，积极推进城市设计工作和规划控制数字平台建设。按照中、省《新型城镇化规划》对西安的功能定位，以及我市“城市北跨渭河发展”的战略目标，抓住省上启动新一轮渭河治理三年行动计划的契机，加快实施渭河西安段综合改造提升工程，为将来把渭河打造成城中河奠定基础。同时，要依托高陵新型城镇化试点和阎良、临潼副中心城市建设，加快渭河以北地区新型城镇化步伐，力争用5年左右的时间把渭北工业区打造成产城融合、宜居宜业的现代新城。

进一步提升城镇化质量。按照以人为本、四化同步、优化布局、生态文明、文化传承的总体要求，编制出台我市《新型城镇化实施方案》，完善城镇空间开发管制约束性规划体系，全面划定开发建设边界，合理确定用地结构，严格耕地保护，有效保障城镇化健康有序发展。继续深入实施主城区“南优、北拓、东延、西联”战略，加快常宁新城建设，推进幸福林带、纺织城、铁路北客站等七大城市片区改造提升，适时启动东关地区综合改造工程，年内完成10个老旧小区更新改造和20个棚户区改造项目征收任务，回迁安置群众1.9万人。大力提升城乡公共服务一体化水平，启动建设120个农村片区化中心社区和20个标准化社区，年内接收有条件的农村居民进城落户14万人，全面完成剩余的122个新农村重点村建设、3.3万移民搬迁、12万贫困人口脱贫和11.6万农村人口饮水安全任务，在全省率先实现四个“清零”。

进一步完善城市基础设施。探索建立缓堵保畅长效化机制，不断优化提升城市道路承载能力。全面启动地铁1号线（二期）建设，年内3号线进入设备调试阶段，4号线土建工程完成过半，加快纺织城至临潼市域轻轨和火车站改扩建工程进度。启动实施西铜一级公路渭河以南段市政化改造，年内完成长乐东路、东南二环立交（二期）等13项城市道路交通工程，继续改造、优化、打通一批城市道路交叉口、拥堵点和断头路，新建公共停车位8000个、新能源汽车充电桩500个。协力推进西咸北环线等3条省市共建高速公路项目，加快国道310、108和省道107等干线公路改扩建工程。大力推进公交都市建设，进一步提高轨道交通、常规公交、慢行交通的接驳能力，年内新购公交车800辆、投放公共自行车1万辆，公共自行车服务网点实现主城区全覆盖。着力增强城市保障能力，强力推进地下排水管网改造、架空线网落地等重点基础设施项目，开工建设引蓝济李、大峪引水等一批水利工程，新增城市日供水能力30万吨、集中供热面积1200万平方米、变电容量100万千伏安。

（六）综合施策创宜居，大力推进城市治理现代化。

创新城市管理体制机制。按照事、责、权、利相统一的原则，进一步赋权于区，推动日常市场监管和执法重心下移，切实解决“看到管不到，管到看不到”的问题。全面推行城市网格化管理，积极整合单元网格的市容、环保、综治等管理职能，加快建立一体化、全覆盖的城市综合管理数字平台，形成责权明确、智能高效的城市精细化管理新模式。

深入推进美丽西安建设。全力打好治污减霾攻坚战和持久战，年内拆除燃煤锅炉150台，淘汰黄标车及老旧车1.5万辆，确保实现主城区20蒸吨以下燃煤锅炉、煤场、居民蜂窝煤用户和全市党政机关事业单位黄标车四个“清零”，全年优良天数达到180天以上。巩固提升“两河一渠”治理效果，不断提高城市污水处理能力，年内新建和改扩建污水处理厂11个。统筹推进八水润西安工程，全面完成浐河上游段堤防和溢流坝工程，加快泾河、石川河等中小河流治理，推进涝渭、黑渭、泾渭等湿地建设，新增湿地6350亩、生态水面2800亩。继续以坚决的态度、强有力的措施，依法保护秦岭生态环境，突出抓好河道景观提升、服务驿站、数字秦岭等6项重点工程，探索建立周至老县城生态保护示范区，全力打造秦岭生态金字招牌。深入推进国家生态园林城市和森林城市创建工作，加快建设汉长安城遗址公园，继续实施屋顶绿化、立体绿化和城改边角地绿化，推动建成一批城市林带，全年新增城市绿地面积400万平方米，完成造林7.5万亩。加快美丽乡村建设，年内创建市级以上生态镇村15个。

加强基层治理能力建设。进一步加大老旧社区服务设施的改造力度，健全社区服务体系，年内实现城市社区组织管理盲点动态“清零”。依法做好村委会换届工作。加强社会组织培育和管理，建立志愿服务长效机制，完善政府购买公共服务机制，促进政府治理与社会自我调节、居民自治良性互动。健全有效预防和化解社会矛盾的体制机制，建立村、社区法律顾问制度，组建专职人民调解员队伍，提升流动人口和特殊人群服务管理水平，扎实做好社区矫正工作。

全方位构筑城市安全“防火墙”。完善立体化社会治安防控体系，有效防范和打击各类违法犯罪活动。巩固安全生产“三化”建设成果，切实加强对地铁、高层建筑、特种设备、市政公用管线等的安全管理，坚决遏制重特大安全事故发生。积极创建国家食品安全城市，建立覆盖从生产加工到流通消费全过程的最严格监管制度，启动建设西安食品药品检验检测中心，年内改造提升“小餐饮”6000户。深入实施质量强市战略，年内建成西安特种设备安全运行监控中心，确保通过全国质量强市示范城市验收。全面提高城市防灾避险能力，切实保障人民群众生命财产安全。

（七）多措并举惠民生，扎实办好“十项惠民实事”。

推动实现更高质量的就业。完善促进就业创业的政策体系，加强就业政策与宏观经济政策及财税、金融、产业等政策的衔接配合。坚持把吸纳高校毕业生就业摆在更加突出的位置，统筹做好农村富余劳动力转移就业和城镇就业困难人员就业帮扶工作，着力提升就业援助服务精准化水平，年内实现全市零就业家庭动态“清零”。推动创业孵化基地建设，以创业带动就业。全年实现城镇新增就业11万人，农村劳动力转移就业65万人。

切实增强社会保障能力。进一步改善城区困难群众的生活，扎实做好城乡居民分类施保工作，适时提高困难家庭临时救助标准和医疗救助报销比例，做到托底线、保基本、救急难。加快完善社会保险关系转移接续和参保缴费政策，推进“五险合一”经办服务标准化建设，不断提高各项社会保险待遇水平。突出抓好“两房并轨”制度的落实，进一步完善保障房周边基础配套及公共服务设施，年内新开工保障性安居工程12万套，提供房源3万套。积极推进养老服务综合改革试点，加快养老服务设施建设，力争全市养老床位达到老龄人口的30‰以上。

推动各项社会事业协调发展。加快实施第二期学前教育三年行动计划。全面完成浐灞丝路学校等36所开发区公办学校建设，改造提升不少于50所原有学校，确保年内投入使用。启动181所义务教育学校标准化建设，完成普通高中教育质量提升工程，促进优质教育资源均衡配置。进一步深化县区级公立医院综合改革，完善医疗联合体和医疗托管模式，建立健全分级诊疗制度。加快市人民医院、市第三医院建设进度，5月底前市胸科医院、市精神卫生中心投入使用，年内完成100个标准化示范村卫生室建设。进一步完善基层公共文化体育设施，新建和更新全民健身路径、农民体育健身工程各200个，完成惠民演出1000场。深入开展精神文明创建和双拥共建活动，进一步加大对残疾人事业的支持力度，继续做好民族、宗教、参事、人防、气象、史志、档案等工作。

四、深入推进依法行政加快建设法治政府

紧紧围绕中央“四个全面”的战略部署，认真落实市委关于全面推进法治西安建设的实施意见，加快建设职能科学、权责法定、执法严明、公开公正、廉洁高效、守法诚信的法治政府，不断提高政府治理现代化水平。

严格依法履行政府职能。按照“法无授权不可为、法定职责必须为”的原则，加快推进政府机构、职能、权限、程序、责任法定化，坚决杜绝法外设权、法外用权。在继续简政放权的同时，更加重视履行公共服务、市场监管、城市治理等职责，切实解决好与群众直接相关的食品安全、环境污染、物业管理等突出问题。

积极推进政府运行机制法治化。严格重大决策合法性审查，加强咨询论证、风险评估、责任追究等关键环节的法制保障，确保决策制度科学、程序正当、过程公开、责任明确。加快完善政府规章制定程序，拓宽公民有序参与政府立法途径，坚决杜绝部门利益法律化现象。进一步规范行政执法行为，积极推进综合行政执法，着力解决权责交叉、多头执法、执法不作为慢作为乱作为等问题，切实提高执法水平。

不断强化对行政权力的制约和监督。依法接受人大及其常委会的监督，自觉接受政协的民主监督，主动接受媒体和公众监督，认真办理人大代表建议和政协提案。扎紧织密权力制约的笼子，对权力集中的部门和岗位，实行分事行权、分岗设权、分级授权、定期轮岗，切实加强行政效能监察，加快推进审计监督全覆盖。按照公开为常态、不公开为例外的原则，全面实行政务公开，重点做好财政预算、公共资源配置、重大项目建设等方面的信息公开，高度重视对网络舆情的回应，努力打造阳光政府。

持续推进政府作风建设。严格遵守党的政治纪律和政治规矩，坚决贯彻中央八项规定和国务院“约法三章”，巩固拓展教育实践活动成果，身体力行“三严三实”和“五个必须”要求，始终做到守纪律、讲规矩。坚持把抓落实的力度和成效作为检验政府工作的“试金石”，全市政府系统各级领导干部都要强化抓落实的主体意识、责任意识和标准意识，勇当抓落实的“主攻手”，决不允许在抓落实上打“白条”、做“虚功”、搞变通，切实做到勤勉敬业、守土有责、守土尽责。坚定不移推进党风廉政建设和反腐败斗争，严格落实“两个责任”，认真履行“一岗双责”，不断深化工程建设、招标投标、土地出让、政府采购、产权交易等领域改革，切实从体制机制上铲除滋生腐败的土壤。进一步加强廉政教育，筑牢思想上拒腐防变的堤坝，努力建设一支勇于改革创新、敢于担当责任、始终勤政为民、严守清正廉洁的公务员队伍。

各位代表！“十三五”是我市全面建成小康社会、全面深化改革开放、全面推进法治西安建设的关键时期，我们要以改革的精神、创新的理念和系统化的思维，科学编制好“十三五”规划，精心谋划一批事关西安长远发展的大项目、好项目，确保我市在未来一个时期保持稳定健康的发展势头。

各位代表！新的一年开启新的希望，新的征程承载新的梦想。让我们紧密团结在以习近平同志为总书记的党中央周围，在市委的坚强领导下，以更实的作风、更高的标准、更大的担当，攻坚克难，锐意进取，努力在新常态下谋求新作为、争创新业绩，不断开创具有历史文化特色的国际化大都市建设的新局面，共同谱写全市人民美好生活的新篇章！
